# MER and increased operative time are not risk factors for the formation of pneumocephalus during DBS

**DOI:** 10.1038/s41598-023-30289-5

**Published:** 2023-06-08

**Authors:** Guglielmo Iess, Giulio Bonomo, Vincenzo Levi, Domenico Aquino, Edvin Zekaj, Federica Mezza, Domenico Servello

**Affiliations:** 1 Department of Neurosurgery, Fondazione IRCCS Istituto Neurologico Carlo Besta, Milan, Italy; 2 Università degli Studi di Milano, Milan, Italy; 3 Neuroradiology Department, Fondazione IRCCS Istituto Neurologico Carlo Besta, Milan, Italy; 4 Department of Neurosurgery, IRCCS Istituto Ortopedico Galeazzi, Milan, Italy; 5 Department of Economics, University of California, Los Angeles, USA

**Keywords:** Parkinson's disease, Movement disorders, Parkinson's disease

## Abstract

Although only recently directional leads have proven their potential to compensate for sub-optimally placed electrodes, optimal lead positioning remains the most critical factor in determining Deep Brain Stimulation (DBS) outcome. Pneumocephalus is a recognized source of error, but the factors that contribute to its formation are still a matter of debate. Among these, operative time is one of the most controversial. Because cases of DBS performed with Microelectrode Recordings (MER) are affected by an increase in surgical length, it is useful to analyze whether MER places patients at risk for increased intracranial air entry. Data of 94 patients from two different institutes who underwent DBS for different neurologic and psychiatric conditions were analyzed for the presence of postoperative pneumocephalus. Operative time and use of MER, as well as other potential risk factors for pneumocephalus (age, awake vs. asleep surgery, number of MER passages, burr hole size, target and unilateral vs. bilateral implants) were examined. Mann-Whitney *U* and Kruskal-Wallis tests were utilized to compare intracranial air distributions across groups of categorical variables. Partial correlations were used to assess the association between time and volume. A generalized linear model was created to predict the effects of time and MER on the volume of intracranial air, controlling for other potential risk factors identified: age, number of MER passages, awake vs. asleep surgery, burr hole size, target, unilateral vs. bilateral surgery. Significantly different distributions of air volume were noted between different targets, unilateral vs. bilateral implants, and number of MER trajectories. Patients undergoing DBS with MER did not present a significant increase in pneumocephalus compared to patients operated without (p = 0.067). No significant correlation was found between pneumocephalus and time. Using multivariate analysis, unilateral implants exhibited lower volumes of pneumocephalus (p = 0.002). Two specific targets exhibited significantly different volumes of pneumocephalus: the bed nucleus of the stria terminalis with lower volumes (p < 0.001) and the posterior hypothalamus with higher volumes (p = 0.011). MER, time, and other parameters analyzed failed to reach statistical significance. Operative time and use of intraoperative MER are not significant predictors of pneumocephalus during DBS. Air entry is greater for bilateral surgeries and may be also influenced by the specific stimulated target.

## Introduction

Deep brain stimulation (DBS) has become an established surgical procedure for the symptomatic treatment of several neurologic and psychiatric disorders such as Parkinson’s disease (PD), essential tremor, dystonia, obsessive-compulsive disorder, epilepsy and Tourette’s syndrome^1–3^.

DBS success greatly depends on different factors, the most important of which is accurate lead placement^4–6^. Although the rapid advances in neuroengineering (e.g. the advent of high-resolution magnetic resonance imaging, the Neuromate robotic system, improved lead anchoring devices) have increased the precision of the procedure, considerable discrepancy across patient’s cohorts still exists^7–9^. Two meta-analyses suggest a 45% rate of lead misplacements with consequent suboptimal therapeutic response^10,11^.

One of the most relevant and debated problems concerning electrode displacement in DBS is represented by the potential intraoperative brain shift caused by cerebrospinal fluid (CSF) leakage following burr hole creation and by consequent air entry in the skull^12^. This phenomenon is called pneumocephalus and, in turn, is thought to provoke unwanted transposition of the brain’s structures.

Some factors such as patient age, cerebral atrophy, multiple microelectrode recordings (MER) passages and use of fibrin glue to cover burr holes may influence CSF loss and brain shift have been examined by different investigators^13,14^. Nevertheless, not much is known whether (and to what extent) operative time has to be considered a significant factor causing air entry during DBS. To date, very few studies have analyzed such correlation, and importantly, they reported conflicting results^13,15^.

Because time is, to some extent, a surgeon-controllable aspect of DBS procedures, if a positive correlation is to be found, this could have an important impact on intraoperative decision-making: use of MER for example, the usefulness of which is still a matter of debate, significantly prolongs mean operative time^16,17^. Although only recently, novel directional lead designs have proven their potential to compensate (at least partially) for sub-optimal electrode positioning, proper lead placement remains the mainstay of this treatment^18–20^.

We present our dataset of 94 patients from two different institutes, composed of 73 and 21 individuals respectively who underwent DBS procedures under stereotactic conditions. Our primary objective was to investigate whether prolonged surgical duration poses the patient at risk of developing pneumocephalus during DBS.

## Material and methods

This is an observational retrospective study which includes a total of 94 DBS procedures, 73 of which performed at IRCCS Istituto Neurologico Carlo Besta (institute 1) from 2014 to 2019 and the remaining 21 at IRCCS Istituto Ortopedico Galeazzi (institute 2) from 2020 to 2021. Data was extrapolated from operative reports and medical records and comprised information such as surgical length of time, patient age, gender and diagnosis, anesthetic protocol (general anesthesia induction vs. awake procedure with only mild sedation and local anesthesia), DBS target, unilateral vs. bilateral surgery, size of the burr hole, use of intraoperative electrophysiological recordings (which included both microelectrode recordings and macrostimulation) and number of MER trajectories.

Pneumocephalus volumes were estimated based on an early postoperative computerized tomography (CT) scan (which was part of the routine practice) at IRCCS Istituto Neurologico Carlo Besta, while were calculated by an intraoperative CT scan at IRCCS Istituto Ortopedico Galeazzi carried out immediately after placing the first (for unilateral procedures) or second (for bilateral procedures) electrodes (i.e. prior securing the definitive leads).

Similarly, it is important to highlight three important differences between the procedures performed at the two institutes (which are described in greater details below): (1) The operative lengths reported in the surgical reports of the two differed for what concerns the starting time: while institute 1 included also time between anesthetic induction and beginning of operation, institute 2 reported solely operative length from the moment of the first skin incision to the final skin suture; (2) The size of the burr holes created to place the electrodes was greater at institute 2 (14 mm vs. 5 mm); (3) While in institute 1 almost 70% of the surgeries required a single MER trajectory, the other center utilized 3 recording tracks for each case.

Surgeries were performed for different neurological and psychiatric conditions; more specifically: Parkinson’s disease, dystonia, essential tremor, obsessive-compulsive disorder, major depression and cluster headache. DBS targets included in the present study were the subthalamic nucleus (STN), the globus pallidus internus (GPI), the ventral intermediate nucleus (VIM) of the thalamus, the posterior hypothalamus, the bed nucleus stria terminalis (BNST), and Brodmann’s area 24 (i.e. the subgenual cingulate gyrus). Informed consent was obtained from all individual participants included in the study. The local ethical committees (IRCCS Istituto Neurologico Carlo Besta, Milan, Lombardia, Italy and IRCCS Istituto Ortopedico Galeazzi, Milan, Lombardia, Italy) approved the study.

**Figure 1:**
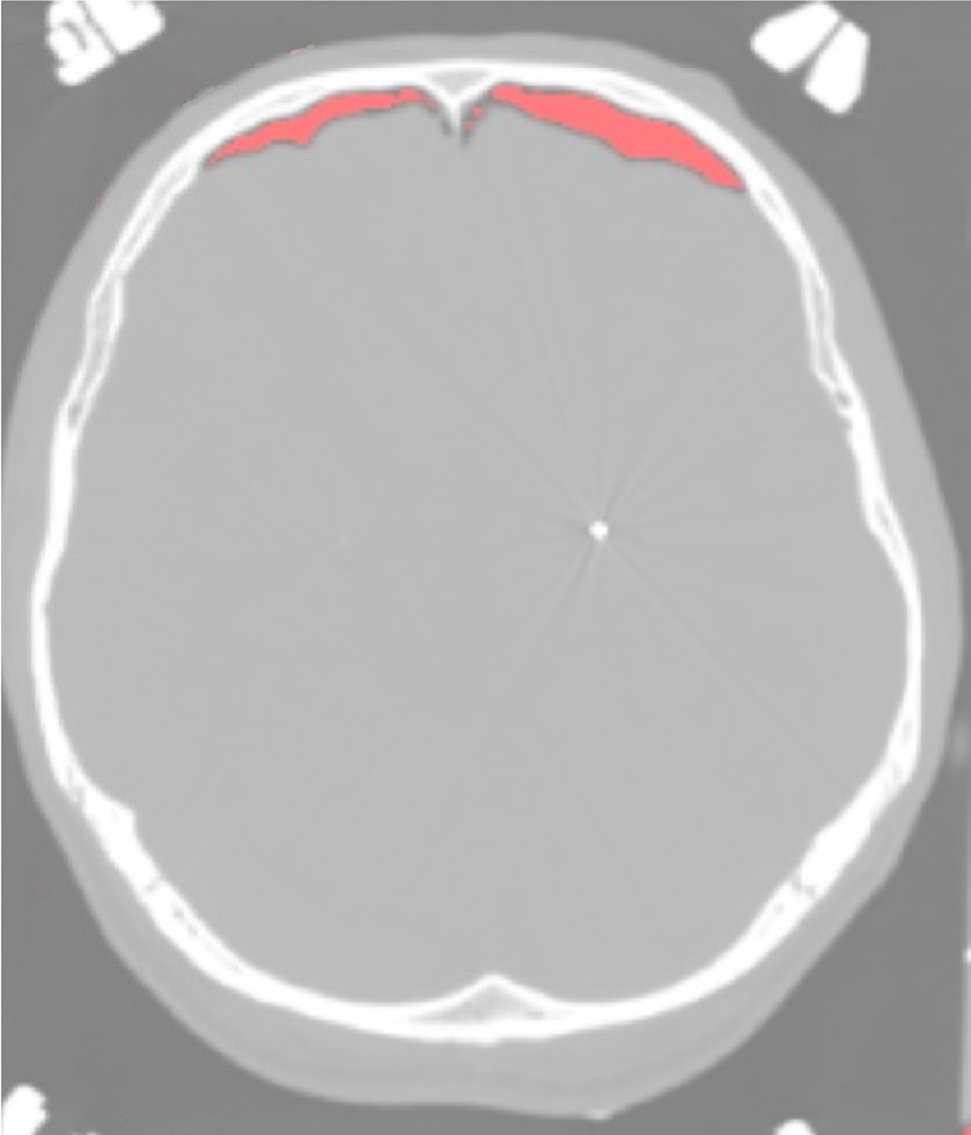
Image illustrating the volume of pneumocephalus calculated with the GUY Matlab method developed at IRCCS Istituto Neurologico Carlo Besta on the postoperative axial-CT images. The area colored in red in the frontal poles represents the intracranial air.

Volumes of pneumocephalus (Fig. 1) were calculated using the following steps: (i) conversion of DICOM CT files in NiFTI format with dcm2nii software (https://people.cas.sc.edu/rorden/mricron/dcm2nii.html). The next steps were implemented in Matlab2017a (www.mathworks.com): (ii) the threshold of the CT NiFTI files was set in order to create a binary brain and skull mask. The threshold is a Hounsfield value above which only the voxels belonging to the brain and skull survive and are set to 1. All values below are set to zero. The threshold was selected empirically for each single case. (iii) At this point the mask is inverted (every voxel of the binary mask is subtracted to 1, i.e. 1-voxel value) to remove skull and brain. In this way the voxels of the air compartment are set to 1. The aim of this mask is to exclude unnecessary voxels from air volume calculation; (iv) The last step requires the voxels of the air volume to be isolated, as other points within the CT volume may have survived to the threshold due to artifacts, noise or other. This was done with a Matlab graphical user interface (GUI) developed at institute 1. Briefly, the GUI allows to select with the mouse one voxel of the previously estimated binary air volume and to isolate it from the other remaining artifactual binary volumes. Figure 1 represents in red the isolated volume; (v) Volume estimation was obtained as the number of the extracted voxels multiplied for the voxel volume.

### Statistical analysis

We used median, interquartile range (IQR), and range to describe continuous variables that were non-normally distributed, and mean and standard deviation (SD) for those that vice versa followed Gaussian distributions, while frequencies and percentages were adopted for categorical variables. We tested continuous variables for normality with the Shapiro-Wilk test and obtained statistically significant values for air volumes, age, and surgical length. Pearson Chi-square test was used to compare frequencies between two categorical parameters, while Mann-Whitney *U* and Kruskal-Wallis tests were employed for comparative analysis of continuous variables between two or more groups, respectively. Partial correlations were utilized to detect associations between two continuous variables, while controlling for other potential confounders.

Because the volume’s scores were non-normally distributed, highly right skewed (1.14 ± 0.25), with many values equal, or close to, zero), and non-remediable by means of mathematical transformations, a generalized linear model with a gamma regression (using a log link function after a +1 transformation) was employed to predict the volumes from 4 dichotomous predictor variables (use of MER, unilateral vs. bilateral surgery, general anesthesia induction vs. awake surgery and burr hole diameter of 5 mm vs. 14 mm), two continuous (age and time), and two non-dichotomous categorical (the “target” variable, which included 6 different brain nuclei/areas and the “trajectory” variable, represented by the use of either one, two, three or more MER trajectories). Of the parameters included in the model, only “target” and “trajectory” were treated as factors, the rest were fitted as covariates. Wald test was utilized for chi-square statistics. A gamma regression was preferred to a linear distribution, a Poisson’, or a negative binomial’ with log link function, based on the more favorable goodness-of-fit measures scores (deviance and Pearson chi-square). The fitted model was compared to an intercept-only model, moreover, resulting in a significant improvement [Likelihood Ratio Chi-square (13) = 31.077, p = 0.003, omnibus test].

Multiple linear regression analysis was employed to predict operative lengths based upon unilateral vs. bilateral surgery, target, age, use of MER, awake surgery vs. asleep procedure, and the institute where DBS was carried. Although the variable “time” presented a modest deviation from a Gaussian distribution [W(94) = 0.972, p = 0.040, Shapiro-Wilk test], the residuals of the multiple linear regression vice versa were normally distributed [W(94) = 0.977, p = 0.103, Shapiro-Wilk test]; therefore, the assumptions of the multiple linear regression analysis were not considered violated. The overall model was compared to an intercept-only model only by means of a F-test, and moreover resulted in a significant improvement [F(6) = 19.262, p < 0.001]. By calculating the variance inflation factors (VIF) between all single independent variables in the regressions of the study, we excluded possible multicollinearity issues in the regression models developed.

All p-values reported are two-tailed and a p < 0.05 was considered statistically significant. Calculations and histograms were made using SPSS (IBM Corp. 2020 Release, IBM SPSS Statistics for MacOs, Version 26.0) and Python (Python Software Foundation 2021 Release, Version 3.8.10 for MacOS).

### Surgical technique at IRCCS Istituto Neurologico Carlo Besta

Surgical procedure is thoroughly described elsewhere^21^. Depending on the target and on the degree of patient collaboration, DBS procedure is performed with the patient either awake or under general anesthesia. The day of the surgery a stereotactic CT scan is performed, and its images are merged with those of a preoperative Magnetic Resonance Imaging (MRI). Final coordinates are calculated on the neuronavigation system (Stealth Station Treon Sofamor Danek, Medtronic Inc. Minneapolis, MN, USA) by adapting the individual patient anatomy to a probabilistic stereotactic digitalized atlas. A small burr hole of 5 mm diameter is drilled, and a rigid cannula is subsequently introduced after opening the dura mater and the arachnoid. The cannula is initially placed 15 mm above the estimated target. At this point of the procedure, to limit cerebrospinal fluid leaks, a fibrin sealant is employed in the burr hole.

If MER are required, they are obtained using a high impedance microelectrode with 0.5-mm steps until 1 mm beyond the target along the single desired trajectory. Based on microrecordings localizing criteria, the definitive electrode (Medtronic Inc. Minneapolis, MN, USA; St. Jude Inc., St. Paul MN, USA) is then positioned at target using the same rigid cannula, after withdrawing the microelectrode.

Bipolar macrostimulation is next briefly performed to test clinical response in the awake patient. Depending on whether MER and macrostimulation show optimal placement, the final electrode is secured using biological glue and with a titanium microplate.

If MER and/or clinical testing suggest an inaccurate electrode placement, a second trajectory is used (using the same burr hole) and the procedure is repeated again. A single trajectory was required in 70% of the DBS procedures, two trajectories in 20% and three or more in 10% of surgeries.

Immediately after concluding the surgical procedure and before returning to the neurosurgical ward, the patient is carried to the unit of neuroradiology to perform a CT scan to exclude postoperative complications. Following the completion of the latter, no surgical decisions are taken (i.e. lead repositioning) based on the amount of intracranial air observed on the postoperative CT scan, apart from conservative measures in cases of symptomatic pneumocephalus (head of bed inclination of 30°, oxygen therapy with high-flow nasal cannula and analgesic therapy).

### Surgical procedure used at IRCCS Istituto Ortopedico Galeazzi

All 21 procedures were performed with the patient awake with only mild sedation. On the day before surgery, a brain MRI is performed which consists in a volumetric gadolinium enhanced T1 sequence and axial T2 or DPI images. On the day of surgery after positioning the CRW stereotactic frame, the patient undergoes a stereotactic CT scan. MRI and stereotactic CT scan images are transferred to the Brainlab Neuronavigation System and subsequently fused together. At this point a direct targeting strategy based on MRI images is performed. The patient is therefore positioned in the operating table integrated with the AIRO system with the head fixed on the CRW frame components. The operating table is shifted inside the AIRO, by applying table movements in order to set up the patient in the most comfortable position. Thereafter, a transparent drape is positioned and anchored with medical adhesive tapes to the AIRO. The frame ring is fixed, and the surgical procedure begins.

A linear skin incision and a 14-mm burr hole created with a high-speed drill are performed centered on the desired trajectory. A 4-mm diamond high-speed drill is utilized to drill out the external cranial bone and to fashion an optimal allocation for the burr hole cap.

Absorbable hemostat, bone dust and fibrin glue are used to seal the burr hole after introducing the rigid cannula. Three microelectrodes are used for MER and advanced 0.5 mm every 30 seconds starting 10 mm above the target and penetrating 1-3 mm below. After completing micro recordings, macro stimulation is performed in order to observe for any eventual adverse events or signs of clinical improvement. After choosing the optimal target, the lead is positioned and fixed with a burr hole cap. Once electrodes are positioned, an intraoperative CT scan is obtained with the aid of AIRO with the patient in a horizontal position and a gait entry of 0°. Lasers of the AIRO are used to evaluate the cranio-caudal and medio-lateral extension of the CT and the first and final acquiring positions are registered. At this point a scout image is obtained to confirm the correct extension of intraoperative CT scan images. If the scout image is in a suboptimal position, the initial and final positions are acquired again until the scout includes the area of interest. At this point the intraoperative CT scan is completed. The images are transferred to the Neuronavigation Brainlab’s software where they are fused with pre-operative images. If an error higher than 2 mm is noted, the leads are repositioned. Similarly to institute 1, no surgical decisions were made based on the amount of intracranial air observed on the intraoperative CT scan. Moreover, none of the 21 DBS procedures of institute 2 required lead repositioning.

### Ethical approval

All procedures performed in studies involving human participants were in accordance with the ethical standards of the institutional and/or national research committee and with the 1964 Helsinki declaration and its later amendments or comparable ethical standards.

### Informed consent

Informed consent was obtained from all individual participants included in the study.

## Results

Overall descriptive statistics of the entire dataset are reported in Table 1 and discussed below, while those calculated separately of institutes 1 and 2, are illustrated more in detail in Tables 2 and 3 respectively.

**Table 1:** Table illustrating the descriptive statistics of the entire case series.

Parameter	Use of MER	Without use of MER
Gender, n [%]	34 females [45.9%]; 40 males [54.1%]	8 females [40%]; 12 males [60%]
Age, years [median; IQR]	54.50; 20	55.50; 26
Unilateral vs. bilateral, n [%]	9 unilateral [12.2%]; 65 bilateral [87.8%]	11 unilateral [55%]; 9 bilateral [45%]
Awake vs. asleep, n [%]	56 awake [75.7%]; 18 asleep [24.3%]	12 awake [60%]; 8 asleep [40%]
Trajectories, n [%]	31 one [41.9%], 15 two [20.3%], 28 three or more [37.8%]	20 one [100%]
Target, n [%]	42 STN [56.8%]; 26 GPI [35.1%]; 3 VIM [4.1%]; 2 HPT [2.7%]; 1 SCG24 [1.4%]	2 STN [10%]; 1 GPI [5%]; 13 VIM [65%]; 3 BNST [15%]; 1 SCG24 [5%]
Volume, cm^3^ [median; IQR]	17.58; 33.94	1.43; 20.84
Time, min [median; IQR]	155; 49	71.50; 78

*STN* subthalamic nucleus, *GPI* globus pallidus internus, *VIM* ventral intermediate nucleus, *HPT* hypothalamus, *BNST* ben nucleus stria terminalis, *SCG24* subgenual cingulate gyrus 24, *IQR* interquartile range.

**Table 2.** Table with descriptive statistics of the case series of institute 1.

Parameter	Use of MER	Without use of MER
Gender, n [%]	23 females [43.4%]; 30 males [56.6%]	8 females [40%]; 12 males [60%]
Age, years [median; IQR]	50; 24	55.50; 26
Unilateral vs. bilateral, n [%]	7 unilateral [13.2%]; 46 bilateral [86.8%]	11 unilateral [55%]; 9 bilateral [45%];
Awake vs. asleep, n [%]	35 awake [66%]; 18 asleep [34%]	12 awake [60%]; 8 asleep [40%]
Trajectories, n [%]	31 one [58.5%], 15 two [28.3%], 7 three or more [13.2%]	20 one [100%]
Target, n [%]	30 STN [56.6%]; 17 GPI [32.1%]; 3 VIM [5.7%]; 2 HPT [3.8%]; 1 SCG24 [1.9%]	2 STN[10%]; 1 GPI [5%]; 13 VIM [65%]; 3 BNST [15%]; 1 SCG24 [5%]
Volume, cm^3^ [median; IQR]	3.53; 37.75	1.43; 20.84
Time, min [median; IQR]	147; 53	71.50; 78

*STN* subthalamic nucleus, *GPI* globus pallidus internus, *VIM* ventral intermediate nucleus, *HPT* hypothalamus, *BNST* ben nucleus stria terminalis, *SCG24* subgenual cingulate gyrus 24, *IQR*, interquartile range

**Table 3:** Table with descriptive statistics of the case series of institute 2.

Parameter	
Gender, n [%]	10 females [52.4%]; 11 males [47.6%]
Age, years [median; IQR]	62; 12
Unilateral vs. bilateral, n [%]	2 unilateral [9.5%]; 19 bilateral [90.5%]
Target, n [%]	13 STN [57.1%]; 8 GPI [42.9%]
Volume, cm^3^ [median; IQR]	25.90; 23.55
Time, min [mean ± SD]	177.43 ± 8.55

Differently from the other center, all cases underwent MER with 3 tracks, and were performed with the patients awake. *STN* subthalamic nucleus; *GPI* globus pallidus internus; *IQR* interquartile range; *SD* standard deviation.

**Figure 2:**
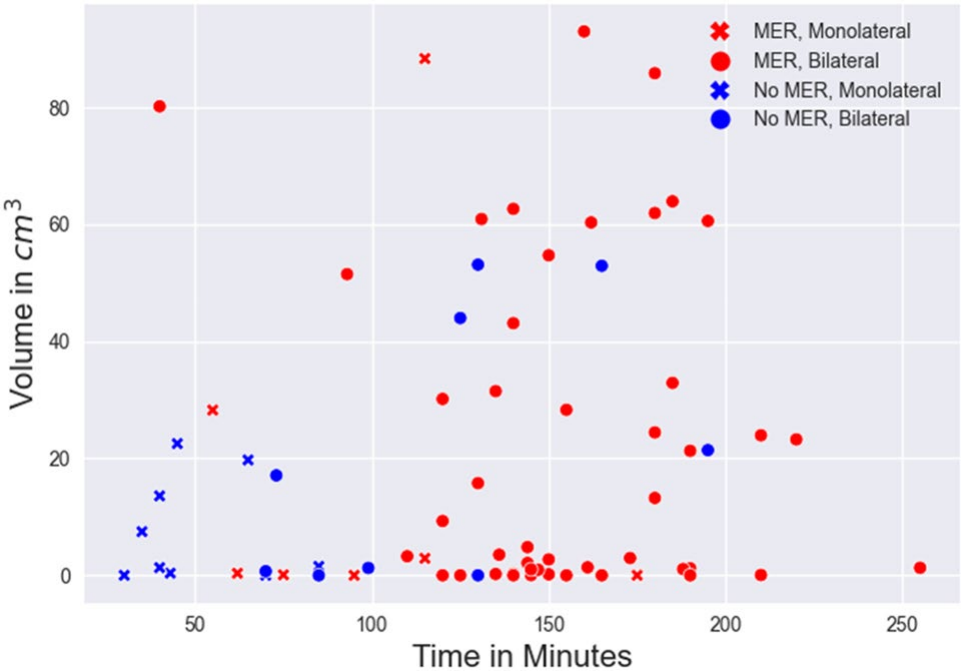
Scatterplot representing surgical length (in minutes) vs. intracranial volume of air (in cm^3^) with labels to identify unilateral vs. bilateral surgeries and eventual use of intraoperative microelectrode recordings.

**Figure 3:**
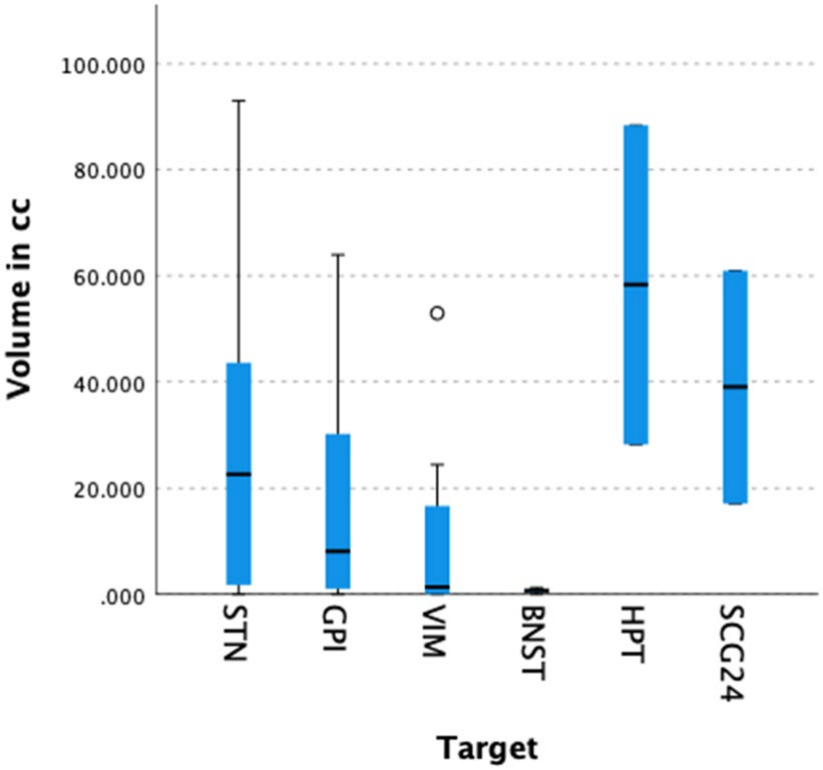
Boxplot describing the distribution of the volumes of air among the different DBS’ targets. *STN* subthalamic nucleus, *GPI* globus pallidus internus, *VIM* ventral intermediate nucleus, *HPT* hypothalamus, *BNST* ben nucleus stria terminalis, *SCG24* subgenual cingulate gyrus 24.

Overall study’s dataset consisted of 42 females and 52 males. Median patient’s age was 54.50 years (IQR = 21, range 8-73), while that of intracranial air volume 13.40 cm^3^ (IQR = 32.10, range 0-93.03). 74 subjects (78.7%) had bilateral electrodes implantations, while 20 (21.3%) had unilateral ones. Of note, bilateral implants had a significantly higher median volume (18.24 vs. 1.43 cm^3^) compared to unilateral implants (U = 497, p = 0.02, Mann-Whitney *U* test, Fig. 2). There was a significant difference between the distributions of volume at the two centers (U = 1056, p = 0.009, Mann-Whitney U test), between the different surgical targets [H(5) = 13.93, p = 0.016, Kruskal-Wallis test, Fig. 3] and between the use of 1 vs. 2 vs. 3 or more MER trajectories [H(2) = 13.039, p = 0.001, Kruskal-Wallis test].

**Figure 4:**
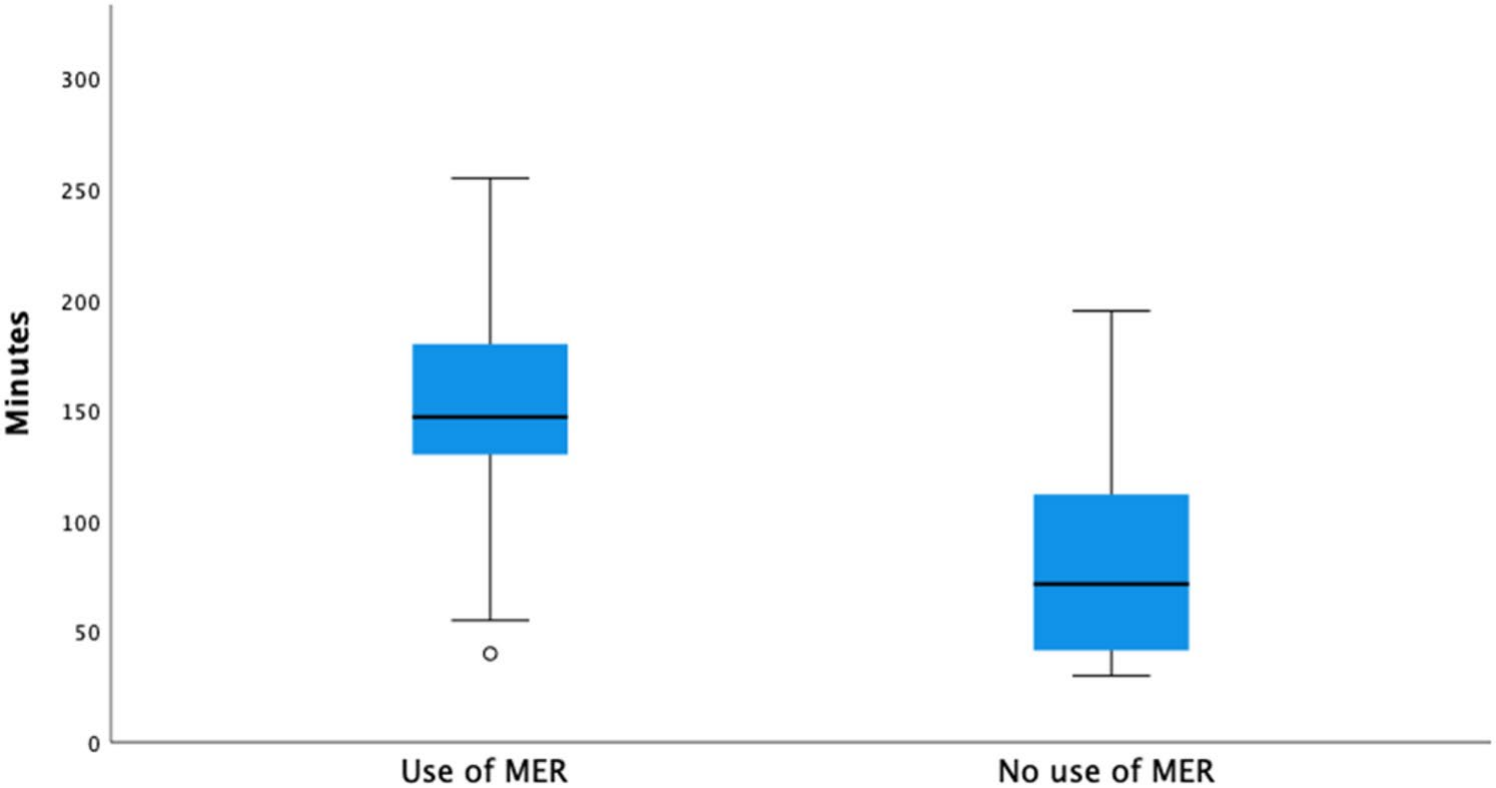
Boxplot illustrating the different distributions of pneumocephalus between patients operated with the use of intraoperative microelectrode recordings, and those operated without. *MER* microelectrode recordings.

Median operative time was 144.50 minutes (IQR = 66, range 30–266) and, as expected, was significantly higher in cases where recordings were obtained (U = 187, p < 0.001, Mann-Whitney *U* test, Fig. 4), for bilateral implants vs. unilateral (U = 64.50, p < 0.001, Mann-Whitney *U* test), and for procedures carried out with the patient awake, compared to the ones performed under general anesthesia (U = 652, p = 0.05, Mann-Whitney *U* test). Nonetheless, after performing multiple linear regression to assess the effects of unilateral vs. bilateral surgery, MER, awake vs. asleep and target on time, only laterality of surgery (B = − 54.05; 95% CI − 74.95, − 33.15; p < 0.001) and MER (B = − 38.66; 95% CI − 61.38, − 15.94, p = 0.001) were confirmed as significant predictors of operative length.

Although subjects undergoing MER presented a slightly higher volume of intracranial air on post-operative CT than those operated without (17.58 vs. 1.43 cm^3^), such comparison did not reach statistical significance (U = 542,00, p = 0.067, Mann-Whitney *U* test).

Moreover, it is important to note that in bilateral DBS 87.8% (65/74) patients had intraoperative MER while only 45% (9/20) with unilateral surgeries were tested with recordings [Χ^2^(1) = 17.25, p < 0.001, Chi-square test].

Furthermore, when controlling for other parameters (unilateral vs. bilateral surgery, target, age, use of MER, general anesthesia vs. awake surgery, burr hole size), time and air volume had a partial correlation of ρ = 0.033, and such value was non-significant (p = 0.758). Similarly, by means of partial correlations, no association was found between age and pneumocephalus (ρ = 0.093, p = 0.389) controlling for unilateral vs. bilateral surgery, target, time, use of MER, general anesthesia vs. awake surgery, burr hole size).

When using generalized linear models (Table 4), unilateral vs. bilateral surgery, and two specific targets (BNST and Hypothalamus) were significant predictors of air entry. The first exhibited a B coefficient of − 1.462 (95% CI − 2.382, − 0.541; p = 0.002), while BNST and Hypothalamus of − 3.356 (95% CI − 5.030, − 1.682; p < 0.001), and 2.312 (95% CI 0.522, 4.102; p = 0.011), respectively. Use of MER, time, number of trajectories, awake vs. asleep procedure, and burr hole size all failed to reach statistical significance.

**Table 4:** Generalized linear model illustrating the effects of various predictor variables on the accumulation of intracranial air.

Parameter	B	95% CI for B	p
STN*			
GPI	− 0.448	− 1.130, 0.235	0.199
VIM	− 0.568	− 1.699, 0.563	0.325
BNST	− 3.356	− 5.030, − 1.682	**< 0.001**
HPT	2.312	0.522, 4.102	**0.011**
SCG 24	0.329	− 1.303, 1.961	0.693
Awake vs. asleep	0.163	− 0.503, 0.828	0.632
Burr Hole size (5 mm vs. 14 mm)	0.024	− 0.976, 1.024	0.962
Age (years)	0.006	− 0.016, 0.027	0.604
Unilateral vs. bilateral surgery	− 1.462	− 2.382, − 0.541	**0.002**
1 trajectory*			
2 trajectories	0.076	− 0.689, 0.840	0.846
3 ore more trajectories	0.586	− 0.333, 1.506	0.211
MER use vs. no MER use	0.649	− 0.346, 1.643	0.201
Time (minutes)	0.000	− 0.007, 0.006	0.909

P values which are statistically significant are evidenced in bold. *MER* microelectrode recordings, *STN* subthalamic nucleus, *GPI* globus pallidus internus, *VIM* ventral intermediate nucleus, *BNST* bed nucleus stria terminalis, *HPT* hypothalamus, *SCG24* subgenual cingulate gyrus 24.

*Set as the reference category.

## Discussion

Brain shift is a well-known phenomenon, and it is now clear that the amount of subdural air entered in the skull during the procedure has a negative impact on the precision of both navigation and stereotactic systems based on preoperative image data^22–24^. Hill et al. calculated a median brain surface shift after incising the dura ranging from 0.3 to 7.4 mm. Other investigators reported a deviation of up to 4 mm shift of subcortical structures^13,25,26^.

Nonetheless, the impact exerted by this loss of accuracy on the clinical outcome is a matter of debate, and is likely determined by the location of the target^14,15,23,25,27^. As brain shift tends to displace targets mainly in the posterior direction, resulting in aberrant pathway activation from pre-operative predicted plan, more posteriorly and deep-located nuclei are affected by a smaller shift compared to anterior and superficial structures^25,27^. Therefore, the position of the specific target in the brain changes the impact that pneumocephalus exerts on the efficacy profile of DBS. For example, while different authors did not report any negative effects due to targeting errors caused by pneumocephalus in patients with PD, a study comparing pathway activation between remitters and non-remitters of subcallosal cingulate DBS in patients with treatment-resistant depression showed how the remitters’ group exhibited a smaller variance in activation of axonal pathways for stimulation^27–29^. Moreover, with a modest mean volume pneumocephalus of 1.77 ± 1.18 cm^3^**,** the same authors found an average shift of the frontal poles of 2.2 ± 1.56 mm^27^. Besides the subcallosal cingulate, other more anteriorly located targets, such as the nucleus accumbens, the anterior limb of the internal capsule (ALIC), and the ventral capsule/ventral striatum (VC/VS), are likely to suffer from a higher degree of displacement compared to the subthalamic nucleus used in PD’s DBS^25,30^. Therefore, in such cases, the long-term efficacy of DBS may be negatively impacted also by smaller amounts of pneumocephalus^14,15,23,25,27^. In any case, high volumes of intracranial air (> 20 cm^3^) have been shown to displace invariably the anterior commissure by 2 mm; therefore it is important that functional neurosurgeons prevent this phenomenon, regardless of the specific target being stimulated^15^.

Although apparently simple in its concept, pneumocephalus is likely a multifactorial phenomenon in which different variables come into play^31^. It is a common belief among surgeons that air influx, rather than being a fast process, takes place over time. As a consequence, longer operative times are thought to come at a price of greater amounts of pneumocephalus^13,14,32–34^. To counteract further air accumulation over time, many centers adopted different strategies like the use of burr hole sealants (like fibrin glue or bone wax), smaller burr hole diameters, saline irrigation, and direct dural puncture to reduce CSF egress^31,35,36^. Yet, although some studies reported a decrease in air inflow, such approaches did not prevent the formation of pneumocephalus^22,31^. This can be explained by the fact that most of the cerebrospinal fluid loss (which is subsequently replaced by subdural gas) is likely to occur immediately after incising the meninges, initially driven by the positive intracranial pressure (ICP) and by the hydrostatic pressure, and thereafter solely by the latter^22,25,37^. Thus, with the patient supine and a neutral head position, after burr hole trepanation and meninges' opening, the volume of CSF subject to outflow is represented by that filling the subarachnoid space extending from the sites of the burr holes to the frontal poles^22^.

Therefore, in line with the concept by which CSF efflux is not a time-dependent phenomenon, we found no evidence of association with operative time by comparing groups of patients undergoing DBS with intraoperative MER (a surgical adjunct which significantly prolongs operative time) with individuals operated by direct image-targeting. However, some publications evaluating pneumocephalus and/or brain shift during DBS did not report any associations with surgical length (although reports accounting for the opposite theory exist as well)^13,15,22,23,25,33,35,38^. Nonetheless, all these studies neglect the need of simultaneously controlling for other potential factors which may also come into play in the formation of pneumocephalus. Among such factors, those most commonly reported in literature include unilateral vs. bilateral surgery, age, burr hole dimensions, number of MER passages, awake vs. asleep procedure, and the specific nuclei being targeted^22,31,35,38^.

To evaluate the effects of operative time on pneumocephalus it becomes therefore essential to perform a multivariate analysis to control for these additional parameters which might act as confounders. For example, in our study bilateral implants presented a higher amount of intracranial air that is statistically significant compared to unilateral’s, but at the same time MER were more frequently employed during bilateral procedures (which not surprisingly were also lengthier surgeries compared to the unilateral ones). After performing generalized linear models two variables emerged as significant predictors of pneumocephalus: side of surgery and type of target. The other parameters included in the regression (length of surgery, MER, age, 14 mm vs. 5 mm burr holes, awake vs. asleep surgery, number of MER passages) did not reach statistical significance.

**Figure 5:**
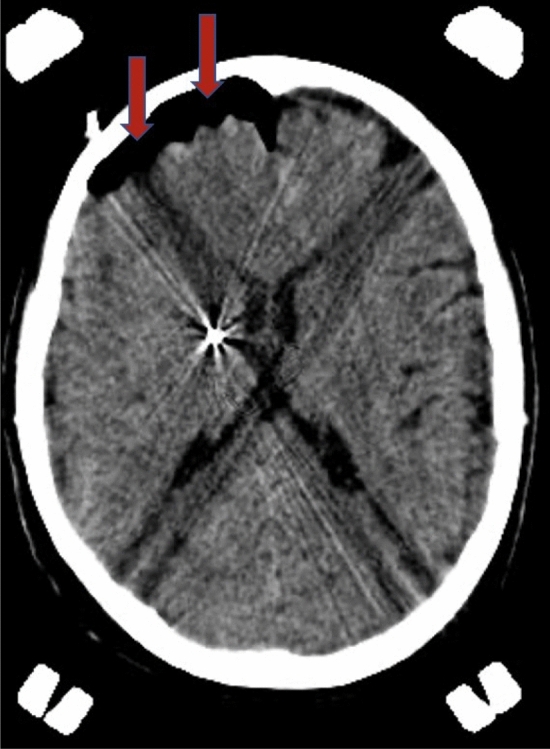
CT scan performed after a unilateral DBS procedure showing the entry of intracranial air (indicated by the red arrows) ipsilateral to the implanted electrode. *CT* computerized tomography, *DBS* deep brain stimulation.

Unilateral implants exhibited a much lower median volume of intracranial air than bilateral (1.43 vs. 18.24 cm^3^). Moreover, such accumulation of air was localized almost invariably along the ipsilateral hemisphere (Fig. 5). From a physical point of view, this is probably due to the falx cerebri which acts as a physical barrier preventing part of liquoral egress from the controlateral side^14^. Such pattern of air collection is important because it determines two specific profiles of brain shift during DBS based on the side operated first: because of the ipsilateral pneumocephalus formation, the first unilateral procedure will generate a force pushing the ipsilateral hemisphere posteriorly; nonetheless, owing to the anteroposterior support exerted by the contralateral hemisphere, the resultant force will be directed both posteriorly and medially, therefore determing a contralateral brain shift. If a second electrode is inserted on the other side, the pressure gradient between the two hemispheres will be neutralized and the previous contralateral shift will reset^14^. In this case, a symmetrical bilateral air invasion takes place, promoting a significant posterior shift of the second target^14,28^. These different patterns of brain shift imply that the first target will be medially and posteriorly shifted, while the second will have a higher grade of posterior shift, but no change in the medio-lateral direction^14,28^.

No differences were noted in our series concerning the amount of intracranial air between patients undergoing awake procedures vs. those under general anesthesia. Few authors have analyzed this issue, and furthermore have reported conflicting results^29,33^. General anesthesia induction can potentially influence air volume in two ways: (1) by shortening surgical procedures, and (2) by enabling an improved control over Valsalva and coughing, maneuvers capable of increasing ICP, and therefore of causing sudden CSF leaks^33^. Our work demonstrated that operative time is not a significant predictor of pneumocephalus. Concerning the latter mechanism of air entry, although our results suggest that a significant are needed to reach more robust conclusions on this problem.

Controversies exist in literature whether the size of the cranial window performed during DBS (and by which CSF exits the skull) must be considered an influential factor or not. This window allows to accommodate multiple MER tracks and is performed by drilling a burr hole through the bone and by opening two layers of meninges (the dura mater and the arachnoid)^22^. Assuming that this parameter is indeed a significant predictor of pneumocephalus, since the surface of the arachnoid opened is limited compared to the one of the bone and of the dura mater (which is usually entirely coagulated and cut), the cleft created by the surgeon in this layer would be the one limiting CSF’s egress.

Importantly, in our study the volumes of intracranial air were significantly higher in patients operated at institute 2 compared to institute 1. Because at institute 1, the burr holes used were smaller (5 mm vs. 14 mm), it could be argued that the wider bone drilling could potentially account for the higher amount of CSF egress. Nonetheless, with multivariate analysis, this parameter failed to display a significant effect on the volume of intracranial air, a finding which is in accordance with other two studies in literature^35,38^.

The reason for which the size of the burr hole does not significantly influence the volume of CSF lost relies on the application of fluid dynamics’ principles, more specifically on Torricelli’s law^39^ which states that the speed of efflux (*v)* of a fluid through an orifice of a container filled to a depth *h* (which, in the case of a DBS procedure with the patient lying supine, is represented by the distance between the burr hole and the frontal pole) is described by the equation $v = \sqrt{2gh}$ (where *g* is the acceleration of gravity). Such law implies that the speed of CSF outflow is not constant and decreases in relation to the amount of CSF that is lost from the skull. Although, in theory, the volumetric rate of CSF loss depends both on the fluid’s speed and on the cross-sectional area of the hole, the speed of efflux is such that most of the CSF outflow takes place during the first seconds after opening the meninges, despite the difference in burr hole diameters^22,25^. In other words, the additional time for CSF’s egress gained by performing a smaller burr hole is too short in relation to the overall duration of the surgical procedure to generate a significant effect on the volume of intracranial air entered. Conversely, with even smaller windows available for CSF efflux (as the one represented by the surface of the arachnoid pierced) another more complex physical property may come into play (i.e. the fluid’s surface tension)^40^, which may potentially limit the volumetric egress of CSF. Because the surface of arachnoid opened was similar between the two institutes, this may explain why the difference between the volumes of air found in univariate analysis was not confirmed in multivariate analysis. The difference yielded using univariate analysis is more likely to be due to other reasons (i.e. confounding effects of other variables) as explained in greater detail below.

Similarly to the burr hole dimensions, also the number of MER trajectories have been an object of debate among researchers^13,23,25,41^. In theory, a higher number of MER passages would require a larger surface of meninges opened, thus an increased volume of CSF leak. Indeed, a simple comparison with the Kruskal-Wallis test seemed to confirm this hypothesis; nevertheless, such parameter failed to reach statistical significance in multivariate analysis. In contrast with our findings, some authors did report an association between the number of tracks used and greater amounts of pneumocephalus (or brain shift)^13,41^. Nonetheless, in their studies additional tracks were positioned during surgeries when MER were considered as suboptimal. Therefore, the causal relationship is difficult to demonstrate, as the additional tracks placed may have been the effect of the previously formed pneumocephalus which was influencing the quality of the recordings. Moreover, other researchers did not find any associations between the two variables^23,25^. In our study the number of MER tracks used by the two institutes was different: while institute 1 started the procedures using a single microelectrode (further tracks were eventually added to improve the localization of the target as needed), institute 2 always used three. Nonetheless, as previously said, the amount of arachnoid opened at both institutes was similar, being tailored to fit potentially multiple MER tracks. Accordingly, an association between the number of trajectories and pneumocephalus would have been difficult to detect.

We are the first to report a significant difference in terms of volumes of intracranial air between targets. More specifically, two targets exhibited a significant difference compared to the STN (which was used as a reference category in multivariate analysis): the bed nucleus of the stria terminalis and the posterior hypothalamus. The former presented significantly decreased volumes of pneumocephalus with respect to the latter. Whereas we did not find any direct explanation for the lower volumes of air of BNST’s DBS, it is important to note that in both cases of hypothalamic stimulation the ventricles were entered. Therefore, it is possible that the penetration of the ventricular system may have led to CSF redistribution through the subarachnoid space and thereafter to its further loss from the burr holes^25^. Such interpretation has been reported in literature also by Khan et al.^25^, although other three papers did not find any associations between pneumocephalus and ventricle penetration^13,15,41^. Because of its specific anatomical location, the hypothalamus is likely to be a target at increased risk of ventricle penetration compared to STN, GPI and VIM. Careful trajectory planning may be of utmost importance to avoid pneumocephalus in such cases. Although such result represents an interesting finding, caution must be exerted because of the small number of cases analyzed.

If the burr hole dimensions and the number of trajectories were not significant predictors in multivariate analysis, why were the volumes of air different between the two centers? A tentative explanation may be given by analyzing the distributions of the two significant predictors (target and unilateral vs. bilateral surgery): while institute 2 included only STN and GPI DBS, institute 2 also performed 16 cases of VIM stimulation (21.9%) which, after BNST, represent the target with the lowest median volume of air (Fig. 3). similarly, 90.5% of the implants were bilateral at institute 2, compared to 75.3% at the other center. Despite these differences we can’t exclude that unidentified dissimilarities between the surgical techniques of the two centers could have affected the results.

Another important problem is the one related to brain atrophy. Since patients with higher degrees of brain atrophy have enlarged subarachnoid spaces (and therefore a higher proportion of CSF/brain parenchyma than their normotrophic counterparts), they may be at increased risk of developing pneumocephalus^41^. In our paper, in order to find an indicator which could account for brain atrophy, we identified age as a first proxy for it; nevertheless, by including this parameter in multivariate analysis, we did not find any significant associations with pneumocephalus^42^. Likewise, other studies utilizing other indirect estimates for brain atrophy (i.e. ventricular volume) failed to report any correlations^14,15^. Only Azmi et al. by assessing the ratio of extra-axial CSF to total intracranial volume, conversely identified a significant association with the amount of air entry; it may be worth adopting this parameter to analyze brain shift during DBS in the future^41^.

As previously stated, since CSF egress is a gravity-dependent phenomenon, the amount of fluid that is lost during surgery is influenced by the site of the cranial trephination, which many centers place slightly anterior to the coronal suture^43,44^. Strictly correlated to the site of the burr hole, the head’s inclination relative to the local vertical (largely determined by gravity) is also essential to assess the amount of CSF subject to outflow, and moreover the direction of the resultant brain shift^13,22,24^. Considering a patient in supine position, after replacing the CSF, air will accumulate at the frontal poles causing a posterior shift of the neural structures along an anterior-posterior axis^13,24^. Thus, besides the site of the cranial trephination, the head’s inclination also becomes an important factor in order to assess the amount of fluid subject to outflow and the force causing the brain shift.

As at both centers the procedures were carried out with the patient in quasi-supine position (usually with only a mild cervical flexion to improve overall comfort) and the burr hole was placed slightly anterior to the coronal suture, it was not possible to assess the effects of the site of the burr hole and of the head’s inclination on the volume of intracranial air. Nonetheless, we believe that planning the surgical procedure placing the burr hole at the highest point of the skull, may be a useful solution to minimize the quantity of air filling the subdural space^12^. Although the position of the burr hole can vary only up to a certain point, it is possible to regulate the inclination of the head by changing the patient’s position. Utilizing a semi-sitting position may be advantageous from this point of view because it relocates the burr hole on top of the skull. In addition to minimizing the quantity of CSF on top of the burr hole, the semi-sitting position changes the orientation of the brain with respect to to the local vertical. Consequently, air would accumulate on top of the skull concavity resulting in a superior-to-inferior force.

### Limitations

Some important limitations to our study must be acknowledged. First of all, the surgical length reported at the two institutes was not uniform: while that of institute 2 corresponds to the time elapsed from skin incision to skin suture, the second included also anesthesiologic time. Since the time for anesthesiologic procedures may be considered marginal during awake surgeries at institute 1 (it included solely the interval needed for patient positioning and local anesthetic injections), it can be regarded as negligible. Conversely, when general anesthesia was required, the operative lengths were more likely to be affected. Therefore, among the independent variables analyzed in our study, we believe that this bias may have been a concern mostly for asleep vs. awake procedures. Although with multivariate analysis a control asleep vs. awake DBS was added, it is possible that such bias may have partially influenced the results. Secondly, we did not examine in our analysis arterial pressure, which some authors consider a potential risk factor for pneumocephalus because of the changes of ICP associated with the cardiac cycle^38^. Lastly, although BNST and hypothalamus emerged as significant predictors of intracranial air, caution must be exerted when interpreting these results because of the low number of corresponding cases analyzed in our dataset.

## Conclusion

Operative time and use of intraoperative microelectrode recordings do not significantly influence the formation of pneumocephalus during deep brain stimulation procedures. In a similar manner, other potential risk factors (i.e. size of the burr holes, number of MER trajectories, patient’s age and awake vs. asleep surgery) are not significant predictors of intracranial air volume. Diversely, bilateral implants are at significantly increased risk compared to unilateral’s of developing pneumocephalus. Target’s location may influence the volume of subdural air (the bed nucleus of the stria terminalis and the posterior hypothalamus may be at lower and at higher risk for pneumocephalus respectively). Minimizing the amount of air entry in the skull during DBS procedures is crucial to guarantee an accurate lead placement. Creating burr holes at the highest points of the skull in relation to patient’s intraoperative position may represent an effective way to achieve this goal.

## Data Availability

Data is available from the Corresponding Author (GI) upon reasonable request.
